# Effect of ASA on the risk of cerebrovascular ischemic events in patients with PFO

**DOI:** 10.1002/acn3.51638

**Published:** 2022-07-27

**Authors:** Liang Xu, Chang Zhou, Xuemei Pan, Jun Zhou, Heng Sun, Tao Xu

**Affiliations:** ^1^ Department of Ultrasound The First College of Clinical Medical Sciences, China Three Gorges University Yichang Hubei Province China; ^2^ Department of Ultrasound The Third People's Hospital of Yichang City Yichang Hubei Province China

## Abstract

**Background:**

Whether atrial septal aneurysm (ASA) increases the risk of cerebrovascular ischemic events in patients with patent foramen ovale (PFO) remains controversial.

**Objective:**

We constructed a detailed meta‐analysis to assess the effect of ASA on risk of cerebrovascular ischemic events in patients with PFO.

**Methods:**

Randomized controlled trials (RCTs) and observational studies (cohort studies and case‐control studies) that compared PFO‐ASA against PFO alone were included. Pooled odds ratios (OR) estimates and 95% CI were calculated using the fixed‐effect and random‐effect models.

**Results:**

Four RCTs and twelve observational studies (five cohort studies and seven case‐control studies) contributed to the meta‐analysis. The pooled results of case‐control studies showed that ASA increased the risk of cerebrovascular ischemic events in patients with PFO (fixed‐effect model: OR = 3.69; 95% CI: 2.67–5.09; *p* < 0.01, random‐effect model: OR = 3.63; 95% CI: 2.51–5.24; *p* < 0.01). However, poole results from RCTs (fixed‐effect model: OR = 1.24; 95% CI: 0.78–1.95; *p* = 0.36, random‐effect model: OR = 1.27; 95% CI: 0.78–2.08; *p* = 0.34) and cohort studies (fixed‐effect model: OR = 1.35; 95% CI: 0.81–2.23; *p* = 0.25, random‐effect model: OR = 1.40; 95% CI: 0.84–2.33; *p* = 0.20) found no evidence. Overall analysis showed that ASA increased the risk of cerebrovascular ischemic events (fixed‐effect model: OR = 2.30; 95% CI: 1.84–2.87; *p* < 0.01, random‐effect model: OR = 2.11; 95% CI: 1.48–3.01; *p* < 0.01). The sensitivity analysis confirmed the stability of all results.

**Conclusions:**

Although case‐control studies support ASA to increase the risk of cerebrovascular ischemic events in patients with PFO, RCTs and cohort studies challenged the credibility. Further prospective studies are needed to confirm the effect of ASA on patients with PFO.

## Introduction

Patent foramen ovale (PFO) has been identified as one of the etiology of unexplained embolism events.^1^, ^2^ However, the incidence of PFO in the general population is 25%, indicating that some PFOs usually have no clinical effect.^3^ Atrial septal aneurysm (ASA) is a pouch‐like structure formed by the expansion of atrial septum. Some studies have shown that PFO patients with ASA or large right‐to‐left shunt have an increased risk of cerebrovascular ischemic events.^4^, ^5^ However, the evidence in some studies is insufficient.^6^, ^7^ In addition, there may be differences between different types of studies. Therefore, the effect of ASA on the risk of cerebrovascular ischemic events in patients with PFO is unclear. We conducted a comprehensive systematic review and meta‐analysis, including randomized controlled trials studies (RCTs) and observational studies (cohort studies and case–control studies), to fully explore the effect of ASA and provide evidence‐based basis for the prevention and treatment of cerebrovascular ischemic events.

## Methods

This meta‐analysis was performed in accordance with the preferred reporting items for systematic reviews and meta‐analyses (PRISMA) statement^8^ (File S1).

### Search strategy

We searched electronic databases of PubMed, Embase, and MEDLINE from inception through March 2022 with no language restriction. Search terms included “patent foramen ovale”, “PFO”, “stroke”, “atrial septal aneurysm”, “antiplatelet therapy”, “anticoagulant therapy”, “medical therapy”, “transient ischemic attack (TIA)”, “TIA”, “recurrent stroke”, “recurrence of embolic events”, and cerebrovascular ischemic events. In addition, the relevant research references were also manually searched to identify potentially eligible studies.

### Study selection and inclusion

The studies enrolled in this meta‐analysis included both RCTs and observational studies (cohort studies and case–control studies) that evaluated effect of ASA on the risk of cerebrovascular ischemic events (stroke or TIA) in patients with PFO. Comparisons between patients with PFO‐ASA and those with PFO alone should be included in these studies. If all patients in one arm of a study had concomitant large shunt, the study was excluded. For RCTs and cohort studies，studies consistent with medical therapy (antiplatelet therapy and/or anticoagulant therapy) as the only preventive measure for recurrent cerebrovascular ischemic events were included in this meta‐analysis. If studies were from multiple publications of the same population, only data from the latest publications were included. Reviews, case reports, cross‐sectional studies, repeated papers, and conference abstracts were excluded in this meta‐analysis. Two reviewers (L.X. and C.Z.) abstracted the data independently according to selection criteria. Any disagreement was resolved by discussion or referral to a third author (X.P.).

### Quality assessment

The quality of studies was assessed according to Cochrane Handbook for RCTs^9^ and Newcastle–Ottawa Scale for observational studies.^10^ Discrepancies were resolved through negotiation.

### Statistical analysis

We conducted meta‐analyses of RCTs and observational studies (cohort studies and case–control studies) according to different study designs. For observational studies, we performed separate analyses for case–control studies and cohort studies. This was done to examine the consistency of results from different study designs. In addition, we pre‐specified subgroup analyses for the three study designs based on neurological characteristics of exposed population and age range of patients. Odds ratios (OR) and 95% confidence intervals (CI) were calculated for each study and pooled values. If zero endpoint events occurred in 1 arm of a study, continuity correction of 1/2 was used. If zero endpoint events occurred in both arms of a study, we did not include them in the meta‐analysis. According to heterogeneity detected, we used a fixed‐effect model (Mantel–Haenszel method) or a random‐effect model (DerSimonian‐Laird method) to calculate pooled OR estimates. In the absence of heterogeneity, the results of fixed effects and random effects models are similar. In the presence of heterogeneity, both models may be biased. Therefore, we conducted interactive tests of the model against the pooled results of RCTs and observational studies. Study heterogeneity was assessed with Cochran Q test and *I* ^2^ test. The *I* ^2^ value is between 0% and 100%, and larger values show increasing heterogeneity. If *I* ^2^ was >50%, heterogeneity was considered significant; If *I* ^2^ was <25%, heterogeneity was considered significant. To explore the stability of meta‐analysis results, sensitivity analysis was performed by excluding each study one by one. Funnel plots were used to test the possibility of publication bias. For all tests, *p* < 0.05 was considered statistically significant. Review Manager 5.3 software (The Cochrane Collaboration, Copenhagen, Denmark) was used for the statistical analyses.

## Results

### Description of included studies

Our initial search identified 628 records. After step‐by‐step selection and search, four RCTs^7^, ^11^, ^12^, ^13^ and 12 observational studies were finally included in the meta‐analysis. The 1observational studies included five cohort studies^6^, ^14^, ^15^, ^16^, ^17^ and seven case–control studies.^5^, ^18^, ^19^, ^20^, ^21^, ^22^, ^23^ The study selection process is shown in File S2. The main descriptions and patient characteristics of the included studies are shown in Table 1. The quality assessment each study is summarized in File S3.

**Table 1:** Main descriptions and patient characteristics of the included studies.

Study	Study design	Exposure population	Control population	Age range of patients	Mean follow‐Up (years)
CLOSURE I 2012	Randomized	CS or TIA with PFO‐ASA	CS or TIA with PFO	18–60	2
PC 2013	Randomized	IS or TIA with PFO‐ASA	IS, TIA with PFO	≤60	4
RESPECT 2017	Randomized	CS with PFO ‐ASA	CS with PFO	18–60	5.9
PICSS 2002	Randomized	IS with PFO‐ASA	IS with PFO	Unlimited	2
Windecker 2004	Cohort	CS with PFO ‐ASA	CS with PFO	Unlimited	4
Wahl 2012	Cohort	CS or TIA with PFO ‐ASA	CS or TIA with PFO	Unlimited	11
Cerrato 2006	Cohort	CS or TIA with PFO ‐ASA	CS or TIA with PFO	18–60	5.3
Mas 2001	Cohort	CS with PFO ‐ASA	CS with PFO	18–55	3.2
CODICIA 2008	Cohort	CS or TIA with PFO ‐ASA	CS or TIA with PFO	Unlimited	2
Nakayama 2019	Case–control	CS with PFO	Non‐CS with PFO	Unlimited	—
Holda 2021	Case–control	CS with PFO	Non‐stroke with PFO	Unlimited	—
Komar 2012	Case–control	CS with PFO	Non‐stroke with PFO	18–59	—
Bayar 2015	Case–control	CS or TIA with PFO	Asymptomatic with PFO	≤55	—
Goel 2009	Case–control	CS or TIA with PFO	Asymptomatic with PFO	Unlimited	—
Natanzon 2003	Case–control	CS with PFO	Non‐CS with PFO	Unlimited	—
Vitarelli 2014	Case–control	CS with PFO	Asymptomatic with PFO	Unlimited	—

CS, cryptogenic stroke; IS, ischemic stroke; TIA, transient ischemic attack; PFO, patent foramen ovale.

### Meta‐analysis of RCTs

**Figure 1:**
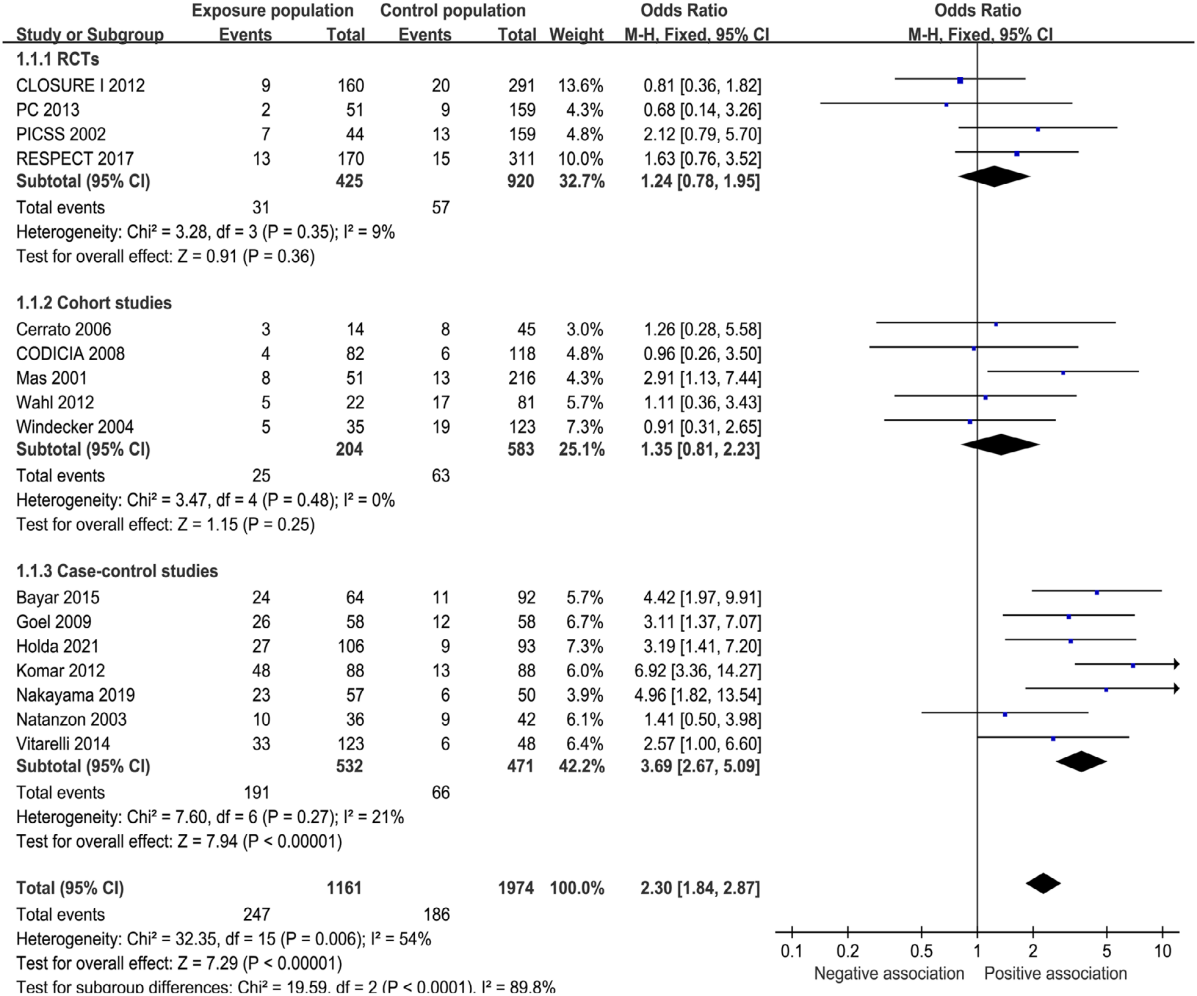
Forest plots comparing the risk of cerebrovascular ischemic events between PFO‐ASA and PFO alone. PFO = patent foramen ovale, ASA = atrial septal aneurysm, RCT = randomized controlled trials, CI = confidence interval. [Colour figure can be viewed at wileyonlinelibrary.com]

Four RCTs were eligible. A total of 425 patients with PFO‐ASA and 920 patients with PFO alone were included in the analysis. The overall incidence of cerebrovascular ischemic events was 7.29% in the patients with PFO‐ASA and 6.20% in the patients with PFO alone. The results of RCTs showed no evidence that patients with PFO‐ASA had an increased risk of cerebrovascular ischemic events compared with patients with PFO alone (fixed‐effect model: OR = 1.24; 95% CI: 0.78–1.95; *p* = 0.36) (Fig. 1). Heterogeneity among studies was not significant (*I* ^2^ = 9%; *p* = 0.35). The results of random‐effect model after interaction test were also not statistically significant (OR = 1.27; 95% CI: 0.78–2.08; *p* = 0.34) (Table 2). Sensitivity analysis showed that the pooled OR ranged from 1.05 (95% CI: 0.59–1.87) to 1.53 (95% CI: 0.88–2.67) after exclusion of the included studies one by one and the overall conclusions remained unchanged.

**Table 2:** Meta‐analysis of RCTs and observational studies.

Study design	No. of studies	Fixed‐effects model			Random‐effects model			Tests of homogeneity	
		OR	95%CI	*p*	OR	95%CI	*p*	*I* ^*2*^ (%)	*p*
All studies	16	2.30	1.84–2.87	<0.01	2.11	1.48–3.01	<0.01	54	<0.01
RCTs	4	1.24	0.78–1.95	0.36	1.27	0.78–2.08	0.34	9	0.35
Observational studies	12	2.76	2.12–3.59	<0.01	2.52	1.74–3.65	<0.01	43	0.05
Cohort studies	5	1.35	0.81–2.23	0.25	1.40	0.84–2.33	0.20	0	0.48
Case–control studies	7	3.69	2.67–5.09	<0.01	3.63	2.51–5.24	<0.01	21	0.27

RCT = randomized controlled trials, OR = odds ratios, CI = confidence interval.

### Meta‐analysis of observational studies

Twelve observational studies were eligible, including five cohort studies and seven case–control studies. For the cohort study, a total of 204 patients with PFO‐ASA and 583 patients with PFO alone were included in the analysis. For the case–control study, a total of 532 patients with PFO‐ASA and 471 patients with PFO alone were included in the analysis. Meta‐analysis of all observational studies showed that patients with PFO‐ASA had an increased risk of cerebrovascular ischemic events compared with patients with PFO alone (29.35% vs. 12.24%; fixed‐effect model: OR = 2.76; 95% CI: 2.12–3.59; *p* < 0.01, random‐effect model: OR = 2.52; 95% CI: 1.74–3.65; *p* < 0.01). Heterogeneity among studies was moderate (*I* ^2^ = 43%; *p* = 0.05). Sensitivity analysis showed that the pooled OR ranged from 2.36 (95% CI: 1.77–3.14) to 2.91 (95% CI: 2.22–3.83) and the overall conclusions remained unchanged.

We performed separate analyses of cohort and case–control studies. In the analyses of cohort studies, we found that although ASA increased the risk of cerebrovascular ischemic events in patients with PFO, the difference was not statistically significant (12.25% vs. 10.12%, fixed‐effect model: OR = 1.35; 95% CI: 0.81–2.23; *p* = 0.25, random‐effect model: OR = 1.40; 95% CI: 0.84–2.33; *p* = 0.20). Heterogeneity among studies was not significant (*I* ^2^ = 0%; *p* = 0.48). Sensitivity analysis showed that the pooled OR ranged from 1.12 (95% CI: 0.62–2.07) to 1.57 (95% CI: 0.90–2.73) and the overall conclusions remained unchanged. In the analyses of case–control studies, ASA increased the risk of cerebrovascular ischemic events in patients with PFO (35.90% vs. 14.86%; fixed‐effect model: OR = 3.69; 95% CI: 2.67–5.09; *p* < 0.01, random‐effect model: OR = 3.63; 95% CI: 2.51–5.24; *p* < 0.01). Heterogeneity among studies was not significant (*I* ^2^ = 21%; *p* = 0.27). Sensitivity analysis showed that the pooled OR ranged from 2.92 (95% CI: 2.04–4.17) to 3.80 (95% CI: 2.67–5.39) and the overall conclusions remained unchanged.

### Overall meta‐analysis

We performed a combined meta‐analysis of RCTs and observational studies. Combined analysis showed that ASA increased the risk of cerebrovascular ischemic events in patients with PFO (fixed‐effect model: OR = 2.30; 95% CI: 1.84–2.87; *p* < 0.01, random‐effect model: OR = 2.11; 95% CI: 1.48–3.01; *p* < 0.01). Heterogeneity among studies was significant (*I* ^2^ = 54%; *p* < 0.01). Sensitivity analysis showed that the pooled OR ranged from 1.92 (95% CI: 1.42–2.59) to 2.30 (95% CI: 1.65–3.19) after exclusion of the included studies one by one and the overall conclusions remained unchanged. The main results of meta‐analysis of RCTs and observational studies are presented in Table 2.

### Major subgroup analyses

We pre‐specified subgroup analyses for the three study designs based on exposure population and age range of patients. In cohort studies, ASA increased the risk of cerebrovascular ischemic events in the subgroup ≤60 years of age (fixed‐effect model: OR = 2.22; 95% CI: 1.01–4.90; *p* = 0.05, random‐effect model: OR = 2.29; 95% CI: 1.03–5.07; *p* = 0.04). In case–control studies, ASA had a significant effect on cerebrovascular ischemic events in the subgroup ≤60 years of age (fixed‐effect model: OR = 5.70; 95% CI: 3.33–9.75; *p* < 0.01, random‐effect model: OR = 5.67; 95% CI: 3.31–9.72; *p* < 0.01). The results of subgroup analysis of RCTs and observational studies are presented in Table 3.

**Table 3:** Subgroup analysis of RCTs and observational studies.

Study design	Subgroup	No. of studies	Fixed‐effects model			Random‐effects model			Tests of Homogeneity	
			OR	95%CI	*p*	OR	95%CI	*p*	*I* ^*2*^ (%)	*p*
RCTs										
	Stroke	2	0.78	0.38–1.60	0.49	0.78	0.38–1.60	0.50	0	0.50
	Stroke or TIA	2	1.79	0.98–3.29	0.06	1.80	0.98–3.31	0.06	0	0.68
	≤60 years' old	3	1.08	0.65–1.82	0.76	1.10	0.65–1.86	0.72	0	0.38
	Unlimited	1	2.12	0.79–5.70	0.13	2.12	0.79–5.70	0.13	NA	NA
Cohort studies										
	Stroke	2	1.64	0.82–3.28	0.16	1.67	0.54–5.24	0.38	61	0.11
	Stroke or TIA	3	1.09	0.52–2.28	0.82	1.09	0.52–2.29	0.82	0	0.96
	≤60 years' old	2	2.22	1.01–4.90	0.05	2.29	1.03–5.07	0.04	0	0.35
	Unlimited	3	1.25	0.65–2.41	0.51	1.29	0.66–2.51	0.45	0	0.44
Case–control studies										
	Stroke	5	3.68	2.49–5.42	<0.01	3.49	2.04–5.98	<0.01	45	0.12
	Stroke or TIA	2	3.71	2.09–6.60	<0.01	3.72	2.09–6.61	<0.01	0	0.55
	≤60 years' old	2	5.70	3.33–9.75	<0.01	5.67	3.31–9.72	<0.01	0	0.42
	Unlimited	5	2.91	1.94–4.36	<0.01	2.89	1.92–4.34	<0.01	0	0.54

TIA = transient ischemic attack, RCT = randomized controlled trials, OR = odds ratios, CI = confidence interval, NA = not applicable.

### Publication bias

**Figure 2:**
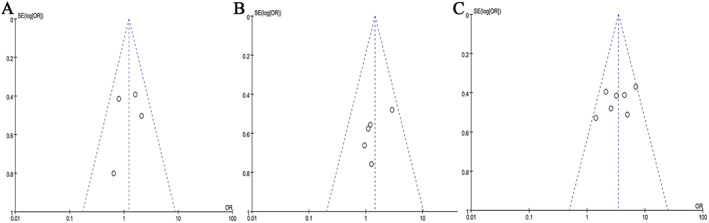
Funnel plots comparing the risk of cerebrovascular ischemic events between PFO‐ASA and PFO alone. (A) RCTs, (B) Cohort studies, (C) Case–control studies. PFO = patent foramen ovale, ASA = atrial septal aneurysm, RCT = randomized controlled trials. [Colour figure can be viewed at wileyonlinelibrary.com]

Funnel plots of cohort studies, case–control studies, and RCTs are shown in Figure 2. For cohort studies, the lower right corner of the funnel, which should include studies with small sample sizes, was missing. For case–control studies and RCTs, funnel plots showed no evidence of publication bias.

## Discussion

Whether ASA can increase the risk of cerebrovascular ischemic events in patients with PFO remains controversial.^4^, ^5^, ^6^, ^7^ Although some studies^15^, ^24^ have shown that patients with PFO‐ASA are the most suitable population for closure therapy, there is insufficient evidence that patients with PFO‐ASA have a higher risk of recurrence than patients with PFO alone. In this meta‐analysis, RCTs, cohort studies and case–control studies were analyzed based on different study designs to help us draw more comprehensive conclusions. Furthermore, we conducted a model interaction test on each of the three pooled results to increase the reliability of results.

Our meta‐analysis showed that the conclusions of RCTs and observational studies were different. The pooled results of observational studies showed that ASA increased the risk of cerebrovascular ischemic events in patients with PFO. However, the pooled results of RCTs showed no evidence that ASA increased the risk of cerebrovascular ischemic events in patients with PFO. Furthermore, the results of separate analysis of cohort and case–control studies were also inconsistent. For cohort studies, although patients with PFO‐ASA had an increased risk of recurrence than patients with PFO alone, the difference was not statistically significant. However，the pooled results of case–control studies showed that ASA significantly increased the risk of cerebrovascular ischemic events in patients with PFO. In our opinion, on the one hand, selection bias and event definition heterogeneity of observational studies will have a certain deviation from the research conclusion. On the other hand, most case–control studies did not use multivariable adjustment methods to adjust for confounding factors, resulting in statistical bias. Third, in RCTs and cohort studies, the low recurrence rate of cerebrovascular ischemic events led to some studies unable to find the difference after follow‐up. Prior to this study, Rigatelli et al.^25^ conducted a meta‐analysis of case–control studies and showed that ASA increased the risk of cerebrovascular ischemic events in patients with PFO (OR: 3.38, 95% CI: 2.72–5.51), which was consistent with the conclusion of our separate analysis of case–control studies. In addition, the current prospective studies on the effect of ASA are not comprehensive, and the recurrence risk of PFO‐ASA is more shown in the subgroup analysis of some studies. To the best of our knowledge，this is the first time to provide a comprehensive analysis of the effect of ASA. In addition, in order to avoid the possible bias of the model in the presence of heterogeneity, we conducted an interactive test of the model, and the conclusion remained unchanged.

Subgroup analysis was performed for each of the three designs according to the factors that might influence the results. Age as influential factor plays an important role in the risk of cerebrovascular ischemic events in patients with PFO. Some studies^3^, ^26^ have shown that the risk of cerebrovascular ischemic events increases with age in patients with PFO. In addition, the neurological characteristics of the exposed population may influence the occurrence of cerebrovascular ischemic events. Therefore, we performed subgroup analysis on the age range included and neurological characteristics of exposed population. The results of subgroup analysis showed that there was significant heterogeneity between different age ranges in cohort and case–control studies. In subgroup analysis of cohort studies, ASA increased the risk of cerebrovascular ischemic events in patients with PFO ≤60 years old. In the subgroup analysis of case–control studies, ASA had a significant effect on patients with PFO ≤60 years old. This is similar to the results of Overell et al.^27^ Overell et al. performed a meta‐analysis and showed that PFO and ASA were significantly associated with ischemic stroke in patients younger than 55 years.^27^ Heterogeneity within comparisons is eliminated by grouping into age bands. As we know, other causes and risk factors of cerebrovascular ischemic events are more likely to play a role in the elderly. Some studies suggest that large shunt may be a high risk factor for patients with PFO.^4^, ^5^ In the study of CLOSE 2017,^4^ all patients with PFO alone were combined with large shunt, may increase the recurrence rate of cerebrovascular ischemic events, resulting in underestimation of the effect of ASA. Therefore, we deleted this study to avoid the potential heterogeneity of RCTs.

Our meta‐analysis had several limitations. First, observational studies included in this meta‐analysis were not adjusted for confounders. Therefore, the effect of ASA on cerebrovascular ischemic events in patients with PFO may be affected by other risk factors. Second, results of meta‐analyses and pre‐specified subgroup data can only be considered exploratory. Third, limitations of non‐randomized studies include selection bias, heterogeneity of event definition, and differences in duration and intensity of follow‐up to events. Fourth, heterogeneity in the definition of ASA was observed in several studies. In most studies, ASA was defined as an atrial septal excursion of ≥10 mm, while some studies used 11 or 15 mm as the cutoff point. The heterogeneity of these definitions cannot be ignored and may affect the results.

## Conclusions

Although case–control studies showed that ASA increased the risk of cerebrovascular ischemic events in patients with PFO, RCTs and cohort studies found no evidence. Further prospective studies are needed to confirm the effect of ASA on patients with PFO.

## Author Contributions

Conceptualization: Chang Zhou and Liang Xu. Data curation: Liang Xu and Chang Zhou. Formal analysis: Xuemei Pan, Liang Xu, and Chang Zhou. Funding acquisition: Jun Zhou. Investigation: Tao Xu, Xuemei Pan, and Heng Sun. Methodology: Liang Xu, Xuemei Pan, and Jun Zhou. Software: Liang Xu and Heng Sun. Supervision: Xuemei Pan and Jun Zhou. Writing—original draft: Liang Xu and Chang Zhou. Writing—review & editing: Chang Zhou and Tao Xu.

## Conflict of Interest

The authors have no conflicts of interest to disclose.

## Supporting information


**File S1.** PRISMA 2009 checklist.

**File S2.** Flow diagram of study selection.

**Table S1.** Risk of bias of included randomized trials.
**Table S2.** Study quality of included comparative observational studies using the Newcastle‐Ottawa scale.
