# Supplementary material for: Effect of ASA on the risk of cerebrovascular ischemic events in patients with PFO
Source: Ann Clin Transl Neurol. 2022 Jul 27;9(9):1384–91. doi: 10.1002/acn3.51638 (PMC9463951; doi:10.1002/acn3.51638)
Supplement: Supplementary file 2 — File S2. Flow diagram of study selection. [file ACN3-9-1384-s002.doc]

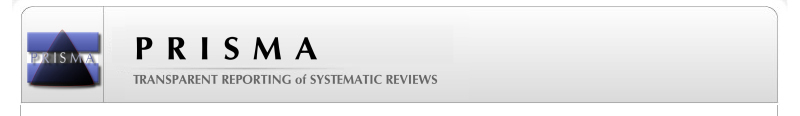
**PRISMA 2009 Flow Diagram**

**Screening**

**Included**

**Eligibility**

**Identification**

Records identified through database searching
(n = 624 )

Additional records identified through other sources
(n = 4 )

Records after duplicates removed
(n = 551 )

Records screened
(n = 551 )

Records excluded
(n = 498 )

Full-text articles assessed for eligibility
(n = 53 )

Full-text articles excluded, with reasons
(n = 25 )

-No related data (n= 10 )

- Low quality (n= 2 )

Studies included in qualitative synthesis
(n = 16 )

Studies included in quantitative synthesis

(n = 16)

-RCT (n=4)

- Cohort studies (n= 5)

- Case-control studies (n=7)
