# Supplementary material for: Effect of ASA on the risk of cerebrovascular ischemic events in patients with PFO
Source: Ann Clin Transl Neurol. 2022 Jul 27;9(9):1384–91. doi: 10.1002/acn3.51638 (PMC9463951; doi:10.1002/acn3.51638)
Supplement: Supplementary file 3 — Table S1. Risk of bias of included randomized trials. Table S2. Study quality of included comparative observational studies using the Newcastle‐Ottawa scale. [file ACN3-9-1384-s001.docx]

**Supplementary Information**

**Effect of ASA on the risk of cerebrovascular ischemic events in patients with PFO**

Running head: Effect of ASA on PFO patients

**Table S1** Risk of bias of included randomized trials.

| Trial | Random sequence generation | Allocation concealment | Blinding of participants and personnel | Blinding of outcome assessment | Incomplete outcome data | Selective reporting | Other bias |
| --- | --- | --- | --- | --- | --- | --- | --- |
| RESPECT 2017 | Unclear risk | Unclear risk | High risk | Low risk | Unclear risk | Low risk | Low risk |
| CLOSURE I 2012 | Low risk | Low risk | High risk | Low risk | Low risk | Unclear risk | Low risk |
| PC 2013 | Low risk | Low risk | High risk | Low risk | Low risk | Low risk | Low risk |
| PICSS 2002 | Low risk | Low risk | High risk | Unclear risk | Low risk | Low risk | Low risk |

**Table S2** Study quality of included comparative observational studies using the Newcastle-Ottawa scale.

| **Included study** | **Year** | **Study quality (Newcastle-Ottawa Scale)** | | | |
| --- | --- | --- | --- | --- | --- |
|  |  | Selection | Comparability | Outcome | Total score |
| Windecker, et al | 2004 | ******** | ***** | ******* | 8 |
| Wahl, et al | 2012 | ******* | ***** | ******* | 7 |
| Cerrato, et al | 2006 | ******* | ***** | ****** | 6 |
| Mas, et al | 2001 | ******** | ***** | ******* | 8 |
| CODICIA | 2008 | ******** | ***** | ******* | 8 |
| Nakayama, et al | 2019 | ******* | ***** | ******* | 7 |
| Holda, et al | 2021 | ******** | ***** | ******* | 8 |
| Komar, et al | 2012 | ******* | ***** | ******* | 7 |
| Bayar, et al | 2015 | ******* | ***** | ******* | 7 |
| Goel, et al | 2009 | ******** | ***** | ******* | 8 |
| Natanzon, et al | 2003 | ******** | ***** | ******* | 8 |
| Vitarelli, et al | 2014 | ******** | ***** | ******* | 8 |
